# Progression from Stage 1 to Stage 3 Type 1 Diabetes Characterized by Hypoglycemia

**DOI:** 10.1210/jcemcr/luaf317

**Published:** 2026-03-12

**Authors:** Aakash Nagarapu, Jamie L Felton

**Affiliations:** Saint Louis University School of Medicine, Saint Louis, MO 63104, USA; Department of Pediatrics, Center for Diabetes and Metabolic Diseases, Indiana University School of Medicine, Indianapolis, IN 46202, USA; Department of Pediatrics, Center for Diabetes and Metabolic Diseases, Indiana University School of Medicine, Indianapolis, IN 46202, USA; Herman B Wells Center for Pediatric Research, Indiana University School of Medicine, Indianapolis, IN 46202, USA

**Keywords:** type 1 diabetes, diabetic ketoacidosis, hypoglycemia

## Abstract

Type 1 diabetes (T1D) development progresses through well-defined stages based on the presence of islet autoantibodies such as insulin autoantibody, glutamic acid decarboxylase autoantibody, tyrosine phosphatase-like protein IA-2 autoantibody, and zinc transporter 8 autoantibody and glycemic status. The presence of multiple islet autoantibodies and normal glucose tolerance is considered stage 1 T1D. Progression to abnormal glucose tolerance, as measured by oral glucose tolerance test or hemoglobin A1c, is considered stage 2, and stage 3 T1D is defined by symptomatic hyperglycemia and the need for insulin replacement therapy. Here, we present a case of a 9-year-old male with rapid progression from stage 1 T1D to stage 3 T1D characterized by persistent, symptomatic hypoglycemia, concurrent with abnormal glucose tolerance. This case demonstrates the paradoxical presence of hypoglycemia and impaired glucose tolerance and suggests that hypoglycemia may be a biomarker of imminent and rapid progression to stage 3 T1D.

## Introduction

Type 1 diabetes (T1D) is characterized by immune-mediated destruction of insulin-producing β cells in the pancreas. The global prevalence of T1D is estimated to be about 8.4 million and is expected to rise to 13.5 to 17.4 million by 2040 [1]. T1D can be predicted months to years before the onset of symptomatic disease by the presence of islet autoantibodies in the blood. This preclinical period progresses along a continuum defined by stage-specific biomarkers: detection of 2 or more islet autoantibodies with normal glucose tolerance is now defined as stage 1 T1D; 2 or more islet antibodies with abnormal glucose tolerance is stage 2 T1D; and clinical symptoms of hyperglycemia (polyuria, polydipsia, unintentional weight loss) and the need for insulin replacement is stage 3 T1D.

In November 2022, the US Food and Drug Administration approved the first immunomodulatory therapy, teplizumab, for delay of stage 3 T1D in high-risk individuals. This anticluster of differentiation 3 monoclonal antibody is given via a 14-day infusion and was shown to delay progression from stage 2 T1D to stage 3 T1D by an average of 3 years, compared to placebo [2, 3]. Though the preclinical stages are well defined, the rate of progression through these stages is highly variable [4]. Birth cohort and longitudinal studies have identified multiple factors that accelerate progression to stage 3 T1D, including higher baseline hemoglobin A1c (HbA1c), tyrosine phosphatase-like protein IA-2 positivity, and younger age [4, 5]. Identification of individuals with stage 1 and stage 2 disease who may be candidates for teplizumab continues to be a challenge. Additionally, the high cost of the drug and the coordination of care required to administer it can prolong the insurance approval process. For individuals who progress rapidly, this means that, at times, the window of opportunity to treat closes before treatment can be initiated. Therefore, further risk stratification for stage 2 individuals is becoming increasingly important.

Progression through early T1D stages is characterized by progressive destruction of insulin-producing β cells and manifests as elevated fasting glucoses, elevated postprandial glucoses, or elevated HbA1c. Hypoglycemia is not classically associated with β-cell destruction in T1D. However, hypoglycemia has been reported in early type 2 diabetes as a result of persistent β-cell dysfunction, and limited reports of hypoglycemia prior to insulin initiation in individuals with T1D suggest that hypoglycemia secondary to dysregulation between α cells and β cells, or dysregulated insulin release, similar to what is observed in hashitoxicosis, may reflect risk of progression in T1D as well [6-9]. Here, we report a case of persistent hypoglycemia in a pediatric patient with stage 1 T1D, followed by rapid progression to stage 3 T1D, and suggest that hypoglycemia may serve as a biomarker of imminent progression to stage 3 in individuals at risk.

## Case Presentation

A 9-year-old male presented to the emergency department (ED) for self-reported hyperglycemia. Over the last year, his mother endorsed progressively worsening polyuria, polydipsia, night sweats, and dizzy spells. Due to these symptoms, she bought a home glucometer. When a random glucose was found to be >400 mg/dL (22.2 mmol/L), she brought the patient to the ED for further evaluation. The patient's mother had been keeping a blood sugar journal over the past year and reported fasting blood glucose was typically 90 to 110 mg/dL (5.00-6.11 mmol/L) but had been trending toward 110 to 130 mg/dL (6.11-7.21 mmol/L) over the past few months. No recent dietary changes or restrictions were reported.

The patient's past surgical history included a perianal fistula and inguinal hernia, repaired at birth and 5 years, respectively. Medical history included chronic constipation managed with lactulose and senna and urinary incontinence managed with mirabegron. He tested positive for COVID in 2020 but did not require hospitalization. Family history is significant for type 2 diabetes in the maternal grandmother but no history of T1D or other autoimmune diseases.

Upon initial presentation, the physical exam was within normal limits with no reported weight loss. No clinical signs or symptoms concerning for diabetic ketoacidosis. A random point-of-care glucose was 147 mg/dL (8.16 mmol/L), and HbA1c was 5.7% (reference range 4.5-5.7%). Complete blood count and complete metabolic panel were unremarkable except for serum glucose of 121 mg/dL (6.72 mmol/L) (reference range 70-99 mg/dL [3.89-5.50 mmol/L]). The endocrinology team was consulted for mildly elevated HbA1c and recommended islet autoantibody testing. Islet autoantibodies were sent, and he was subsequently scheduled for an outpatient oral glucose tolerance test (OGTT) and follow-up in 8 weeks.

However, several days before scheduled outpatient follow-up, he presented to the ED with symptoms of hypoglycemia and a home point-of-care glucose of 30 mg/dL (1.67 mmol/L). The patient appeared sweaty and dizzy. Laboratory testing showed trace ketones on urinalysis and HbA1c of 5.4%. Autoantibody assays from the previous ED visit had revealed the patient was glutamic acid decarboxylase and insulin autoantibody positive, consistent with stage 1 T1D. No exposure to oral hypoglycemic agents, including sulfonylureas, or β blockers was reported. For further assessment of his hypoglycemia, he was admitted for a 24-hour diagnostic fast and critical sample testing. However, glucose did not drop below 51 mg/dL (2.83 mmol/L), and the patient was discharged. Follow-up OGTT was completed several days later with fasting glucose of 79 mg/dL (4.38 mmol/L) and 2-hour glucose of 76 mg/dL (4.22 mmol/L).

The patient presented to the ED for a third time several weeks later with abdominal pain. Glucose was reportedly 57 mg/dL (3.16 mmol/L) at home but had risen appropriately by the time of arrival to the ED. Given the multiple presentations to the ED and concerns for persistent hypoglycemia, the patient was admitted for repeat diagnostic fast and assessment of critical sample testing.

## Diagnostic Assessment

During this admission, a critical sample was obtained when point-of-care glucose dropped to 44 mg/dL (2.44 mmol/L) several hours after starting diagnostic fast. Critical sample laboratory diagnostics, including serum glucose, insulin, C-peptide, GH, cortisol, and β-hydroxybutyrate, were collected, and a glucagon challenge was administered. Results were significant for elevated β-hydroxybutyrate, appropriate GH and cortisol values, and appropriate rise in glucose after glucagon challenge, suggesting concurrent ketotic hypoglycemia and stage 1 T1D (Table 1).

**Table 1.** Critical sample data

Analyte	Result	Reference range
Glucose	48 mg/dL	70-100 mg/dL
	(2.16 mmol/L)	(3.89-5.55 mmol/L)
Insulin	1.46 µIU/mL	4.00-30.00 µIU/mL
	(1.46 mIU/L)	(4.00-30.00 mIU/L)
C-peptide	0.4 ng/mL	1.1-4.4 ng/mL
	(2.86 pmol/L)	(7.85-31.42 pmol/L)
GH	0.656 ng/mL	<0.3 ng/mL
	(4.68 pmol/L)	(2.14 pmol/L)
Cortisol	228.2 µg/dL	8.00-25.00 (µg/dL)
	(6289.41 nmol/L)	(220.48-689 nmol/L)
β-hydroxybutyrate	3.40 mmol/L	0.02-0.27 mmol/L
	(61.26 mg/dL)	(0.36-4.86 mg/dL)

Serum glucose confirmed hypoglycemia (<50 mg/dL, <2.78 mmol/L). Insulin and C-peptide were both low per the reference ranges but inappropriately so given hypoglycemia. This suggests insulin release that is inappropriate or dysregulated but insufficient to prevent ketone formation, as evidenced by an elevated β-hydroxybutyrate. Cortisol and GH are both appropriately elevated in response to hypoglycemia, which rules out adrenal insufficiency and GH deficiency as causes of hypoglycemia.

## Treatment

The patient was started on a continuous glucose monitor and received nutritional counseling for the prevention of ketotic hypoglycemia. Plans were made for further outpatient follow-up and monitoring for early-stage T1D.

## Outcome and Follow-up

**Figure 1.**
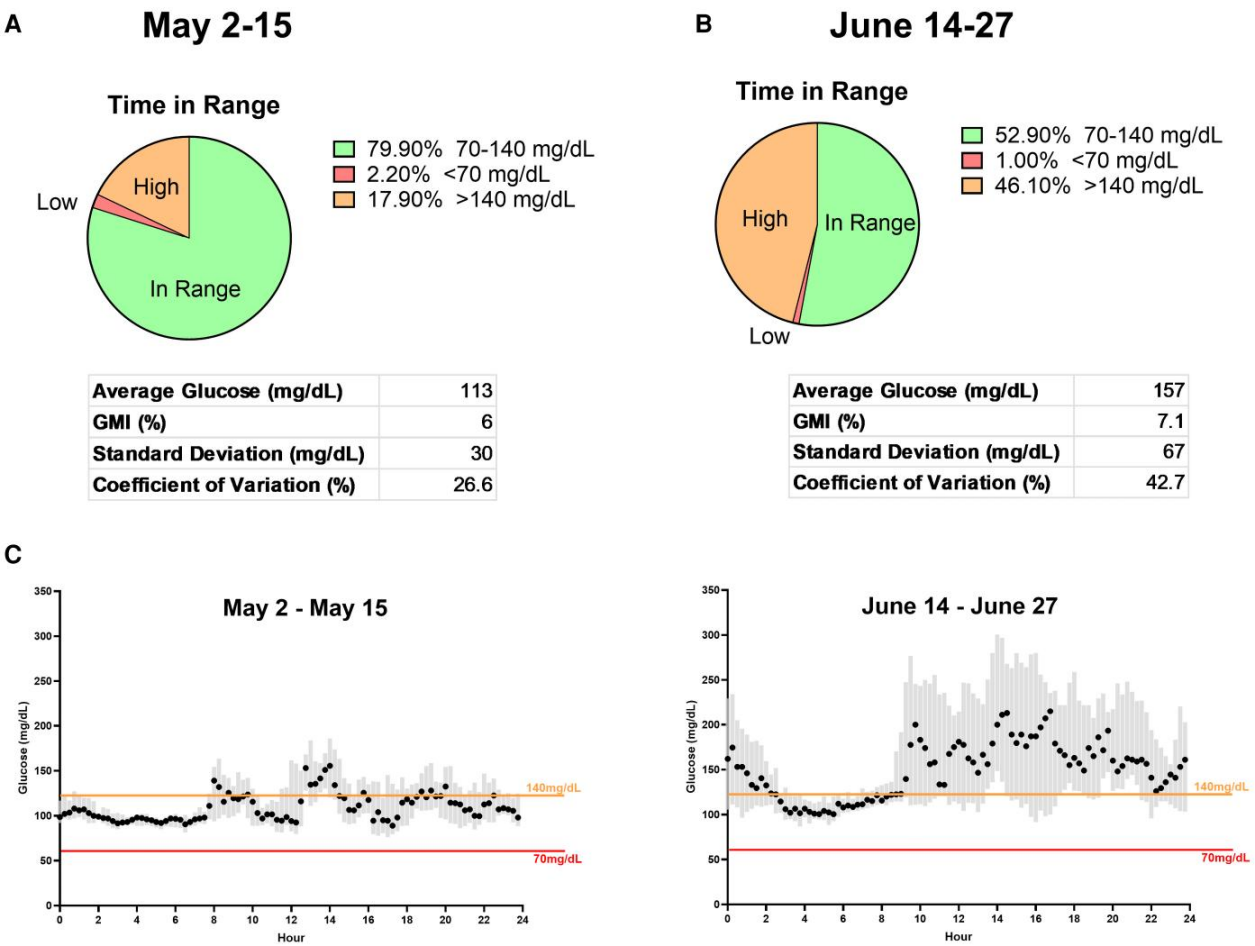
Diabetes biomarkers and labs. Continuous glucose monitoring data were collected using a Dexcom G7 and exported from Dexcom CLARITY®. All tables and graphs were generated by the authors from exported data using GraphPad Prism. Ambulatory glucose profile-style summaries are shown. (A) The initial early stage diabetes clinic visit. The patient was within target glucose range (70–140 mg/dL [SI: 3.89–7.77 mmol/L]) for 80% of recorded values. (B) One month later, time in target range decreased to 53%, with 35% of values above 140 mg/dL ([SI: >7.77 mmol/L]). (C) Side-by-side comparison of glucose distributions over the preceding 2-week periods, demonstrating worsening daytime glycemic control. Median glucose with interquartile ranges is shown.

Four weeks later, the patient was seen as an outpatient where HbA1c was found to be 5.6%. Continuous glucose monitoring data showed an average glucose of 113 mg/dL (6.27 mmol/L) with 80% glucoses within range (70-140 mg/dL [3.89-7.77 mmol/L]) (Fig. 1A and 1C). However, multiple postprandial rises >180 mg/dL (9.99 mmol/L) were noted, and 18% glucoses were >140 mg/dL (7.77 mmol/L). OGTT was not repeated at that time, as it had been completed the month prior and HbA1c had not changed significantly. Plans were made for continued outpatient follow-up and close monitoring for disease progression to stage 2 T1D, at which point he would be a candidate for prevention therapy with teplizumab for delaying onset of stage 3 T1D.

**Figure 2.**
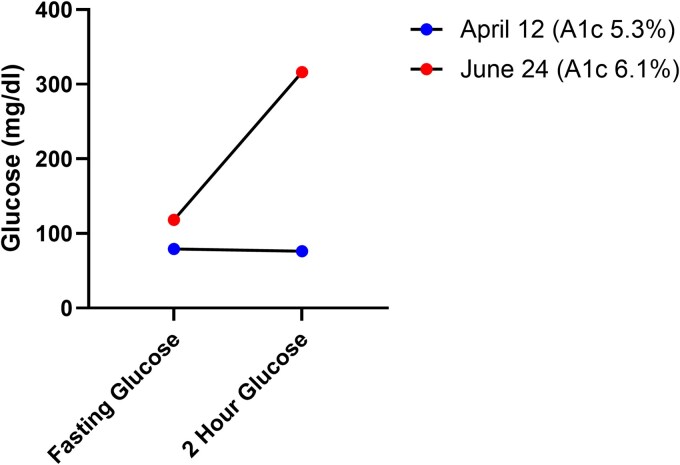
Hemoglobin A1c and oral glucose tolerance test data overtime. Two-hour oral glucose tolerance test results demonstrated dramatic dysglycemic change over 10 weeks, supported by increases in hemoglobin A1c.

Three weeks later, the patient presented to an outside hospital ED with persistent hyperglycemia on continuous glucose monitoring. Per report, glucoses were over 300 mg/dL (16.65 mmol/L) for the last several hours. Basic metabolic panel revealed a normal bicarbonate level and anion gap. Urinalysis was negative for ketones. Review of continuous glucose monitoring data revealed an average glucose of 158 mg/dL (8.87 mmol/L) and only 53% glucoses within range over the last 14 days (Fig. 1B and 1C). During this time, 35% of glucoses were >140 mg/dL (7.77 mmol/L). HbA1c at that time was 6.1% and fasting glucose was 118 mg/dL (SI: 6.55 mmol/L), consistent with stage 2 T1D. However, 2-hour OGTT glucose was overtly dysglycemic at 313 mg/dL (17.37 mmol/L) and met the criteria for stage 3 T1D (Fig. 2). Based on HbA1c, fasting glucose, and multiple islet autoantibody positivity, prior authorization for teplizumab treatment was submitted and approved. The use and efficacy of teplizumab in stage 2 and stage 3 were discussed with the family, who decided to proceed, and the patient received teplizumab infusion 3 weeks later. One year since receiving the infusion, the patient's HbA1c is stable at 6.2% and he is not on insulin.

## Discussion

The ability to predict T1D development based on the presence of multiple islet autoantibodies led to the adoption of a staging system in 2015 [10]. Because progression to T1D is virtually 100% in individuals with multiple islet autoantibodies, their presence alone, with completely normal glucose tolerance, is now considered stage 1 T1D. Progression is characterized by the development of abnormal glucose tolerance based on elevated HbA1c, fasting, or postprandial glucoses. Elevated but not overtly dysglycemic glucoses are considered stage 2 T1D, and overt hyperglycemia with or without clinical symptoms is considered stage 3 T1D and often requires initiation of insulin. The progression throughout these stages is highly variable; however, it is appreciated that the period of time from stage 1 to stage 3 is typically measured in months to years, rather than days to weeks, even in the very young [10, 11].

Here, we present a case of extremely rapid progression from stage 1 T1D to stage 3 T1D that was heralded by persistent and symptomatic hypoglycemia, concurrent with evidence of abnormal glucose tolerance but not overt hyperglycemia. Though rare, this case is consistent with other literature to support hypoglycemia as a potential biomarker for pending rapid progression to stage 3 T1D. A single case report of concurrent fasting hypoglycemia and postprandial hyperglycemia in an autoantibody-positive individual with early T1D led to an analysis of the Fr1da cohort, which screens children aged 2 to 5 years living in Bavaria, Germany, for early-stage T1D [8, 9]. This analysis identified fasting hypoglycemia in a small percentage of autoantibody-positive individuals but not in autoantibody-negative individuals and proposed that fasting hypoglycemia could be an indicator of disease progression. More recently, a retrospective report from Japan identified hypoglycemia in 6.9% of individuals with a T1D diagnosis, prior to insulin initiation [7].

The mechanisms that drive hypoglycemia during autoimmune β-cell destruction remain unclear. Autoimmune thyroid disease progression can be characterized by periods of hyperthyroidism, termed hashitoxicosis, due to the release of preformed hormone during the initial destruction of thyroid follicles [12]. It is plausible that regulation of insulin secretion is disrupted due to lymphocytic infiltration of the islet during early T1D development. At the same time, while α cells are not destroyed during T1D progression, there is significant evidence to support their dysfunction, and failed counterregulation in the early stages of T1D could exacerbate glucose homeostasis and result in both hyper- and hypoglycemia [13, 14]. Further investigation into the factors that drive hypoglycemia in early T1D is required. In summary, this unique case adds to the growing body of evidence for true hypoglycemia during early stages of T1D progression and supports further exploration of hypoglycemia in early-stage T1D as a biomarker for the risk for rapid progression.

## Learning Points

- T1D manifests in various ways in children, but staging has historically been based on evidence of dysglycemia, clinical symptoms, elevated HbA1c, and elevated fasting and postprandial glucose.
- Though dysglycemia in T1D is traditionally hyperglycemic episodes, this unique presentation of hypoglycemic episodes prior to the onset of typical stage 3 diagnosis prompts investigation into the mechanisms behind pathogenesis and progression.
- Our case report highlights the importance of early screening and tracking of glucose levels and responses to garner a high degree of data collection as our patient progressed from dysglycemia to stage 1 and stage 2/3 T1D.

## Data Availability

Restrictions apply to the availability of some or all data generated or analyzed during this study to preserve patient confidentiality or because they were used under license. The corresponding author will on request detail the restrictions and any conditions under which access to some data may be provided.
